# Early vs. Delayed Feeding after Endoscopic Submucosal Dissection for Gastric Cancer: A Systematic Review and Meta-Analysis

**DOI:** 10.3390/medicina56120653

**Published:** 2020-11-27

**Authors:** Jun Watanabe, Joji Watanabe, Kazuhiko Kotani

**Affiliations:** 1 Division of Community and Family Medicine, Jichi Medical University, Shimotsuke-City 329-0498, Tochigi, Japan; m06105jw@jichi.ac.jp; 2 Department of Surgery, Iwami Hospital, Iwami-Town, Tottori 681-0003, Japan; george.watanabe@icloud.com

**Keywords:** diet, endoscopic submucosal dissection, fasting, meta-analysis, stomach neoplasms, patient satisfaction, quality of life

## Abstract

*Background*: Endoscopic submucosal dissection (ESD) for gastric cancer is increasingly performed worldwide due to its efficacy and safety. This study aimed to assess the evidence of the impact of early vs. delayed feeding after ESD on quality of care, which remains to be fully determined. *Methods:* Electronic databases (PubMed, the Cochrane Central Register of Controlled Trials, EMBASE) and the trial registries (the World Health Organization International Clinical Trials Platform Search Portal and ClinicalTrials.gov) were searched for studies performed prior to September 2020. Study selection, data abstraction, and quality assessment were independently performed using the Grading of Recommendations Assessment, Development, and Evaluation approach. Self-rated satisfaction and hospital stay were chiefly analyzed. *Results*: Two randomized controlled trials (239 patients) were included. The early and delayed post-ESD feeding groups had similar rates of post-ESD bleeding (risk ratio 1.90, 95% CI 0.42 to 8.63; I^2^ = 0%). Early post-ESD feeding resulted in increased patients’ satisfaction in comparison to delayed post-ESD feeding (standard mean difference (MD) 0.54, 95% CI 0.27 to 0.81; I^2^ = 0%) and reduced the length of hospital stay (MD −0.83, 95% CI −1.01 to −0.65; I^2^ = 0%). *Conclusion*: Early post-ESD feeding was associated with increased patients’ satisfaction and reduced hospital stay in comparison to delayed feeding, while the rate of complications did not differ to a statistically significant extent. As we must acknowledge the limited number of reviewed studies, various trials regarding the quality of care are further needed to determine the benefits of early feeding after ESD.

## 1. Introduction

Gastric cancer is one of the most common cancers and the third leading cause of cancer death in the world, and approximately one million people are diagnosed with gastric cancer each year, of whom approximately 782,000 die [1,2]. Upper gastrointestinal endoscopy is now widely used for the detection and early treatment of gastric cancer [3]. Endoscopic submucosal dissection (ESD) is an established resection method and is used to remove early stage cancers and large lesions from the gastrointestinal tract [4].

ESD is reported to be associated with a low risk of perforation [5,6,7], short hospital stay [8,9,10], higher quality of life (QOL) [10,11,12], while overall survival is not significantly different from that in patients who undergo gastrectomy for early gastric cancer [10,13,14]. Due to its efficacy and safety, ESD has been increasingly performed worldwide [15]. For patients who undergo ESD, the quality of care, such as hospital stay and QOL, is also important [16]. The timing of feeding after ESD is assumed to contribute to such a care level [17,18,19,20]. Evidence to support the impact of the timing of feeding on the quality of care has not been confirmed, as there have been no meta-analyses and because the relevant guidelines do not include a recommendation [21]. Thus, the present study aimed to compare the quality of care between early vs. delayed feeding in patients after ESD.

## 2. Methods

Our review protocol was registered in protocol.io (dx.doi.org/10.17504/protocols.io.bmejk3cn). The present study was performed in accordance with the Preferred Reporting Items for Systematic Reviews and Meta-Analyses (PRISMA) Statement [22].

Randomized controlled trials (RCTs) were included to assess the efficacy and safety of early vs. delayed feeding after ESD. The early and delayed feeding was defined as the initiation of feeding within 1 day and after 2 days after ESD, respectively, as reported previously [19,20]. All studies were included, including those reported as full text, those published as abstract only, and unpublished data, regardless of language or country restrictions. The inclusion criteria were adult patients (≥18 years of age) who underwent ESD for gastric lesions. The exclusion criteria were residual and recurrent lesions after ESD. The primary outcomes were post-ESD bleeding, patients’ satisfaction, and length of hospital stay (days). Post-ESD bleeding was defined as clinical evidence of bleeding after ESD, as represented by hematemesis, melena, hematochezia after normalization of stool color, a decrease in hemoglobin levels of ≥2.0 g/dL after consecutive stable hemoglobin levels, and/or active bleeding confirmed by endoscopic evaluation. Patients’ satisfaction was defined as the mean score of the numeric rating scale (the lowest score corresponded to the highest level of dissatisfaction, while the highest score corresponded to the highest level of satisfaction). The secondary outcomes were post-ESD ulcer healing, abdominal pain, and all adverse events (perforation, dyspnea, dementia, chest pain). The post-ESD ulcer healing status was defined as a proportion of patients with ulcer stage S1 and S2 by follow-up esophagogastroduodenoscopy at 2 months after ESD [23]. Abdominal pain was defined as the mean score of the numeric rating scale (the lowest score corresponded to the absence of pain, while the highest score corresponded to the highest level of unbearable pain).

The following databases were searched for articles published before September 2020: the Cochrane Central Register of Controlled Trials (CENTRAL), MEDLINE via Ovid, and EMBASE via ProQuest Dialog (Appendix A). The following databases were also searched for ongoing or unpublished trials: World Health Organization International Clinical Trials Platform Search Portal (ICTRP), and ClinicalTrials.gov (Appendix B). The reference lists were checked for studies [3,4,15]. The original authors were asked for unpublished or additional data if necessary.

Two reviewers (Jun W and Joji W) independently screened titles and abstracts, then assessed the eligibility based on the full text. When the relevant data were missing, the original authors were contacted. Two reviewers (Jun W and Joji W) independently performed the data extraction of the included studies using a standardized data collection form, including the information on study design, study population, patient characteristics, and the primary and secondary outcomes. The risk of bias was independently evaluated using Risk of Bias 2 [24]. Any disagreements were resolved by discussion; if an agreement was not reached, a third reviewer (KK) acted as an arbiter. The primary and secondary outcomes, based on the Cochrane handbook, were summarized in a table [25]. The study quality was assessed using the Grading of Recommendations Assessment, Development, and Evaluation (GRADE) approach [26].

We pooled the relative risk ratio (RR) and the 95% confidence interval (CI) for the binary variables. We pooled the mean differences (MDs), the standard MD (SMD), and the 95% CIs for continuous data. When some scales increased with patients’ satisfaction while others decreased, the mean values from one set of studies were multiplied by −1 to ensure that all of the scales pointed in the same direction [25]. All adverse events were described based on the original reports. An intention-to-treat (ITT) analysis was performed for all dichotomous data, whenever possible. For continuous data, missing data were not imputed, based on the recommendation of the Cochrane handbook [25]. A meta-analysis with a random-effects model was performed using the Review Manager software program (version 5.4, The Cochrane Collaboration, Copenhagen, Denmark). Heterogeneity was evaluated by a visual inspection of the forest plots and calculation of the I^2^ statistic (I^2^ values of 0–40%: might not be important; 30–60%: may represent moderate heterogeneity; 75–100%: represents considerable heterogeneity) [25]. When heterogeneity was identified (I^2^ > 50%), the reason for heterogeneity was assessed by a subgroup analysis of lesion size (<20 mm vs. ≥20 mm) when sufficient data were available. As there were less than 10 trials in the analysis, a funnel plot was not performed [25]. The following sensitivity analyses of the primary outcomes were performed: exclusion of studies using imputed statistics, and missing participants, and verification of the robustness of the results by seeking informative missingness odds ratios [27].

## 3. Results

### 3.1. Study Selection and Characteristics

**Figure 1:**
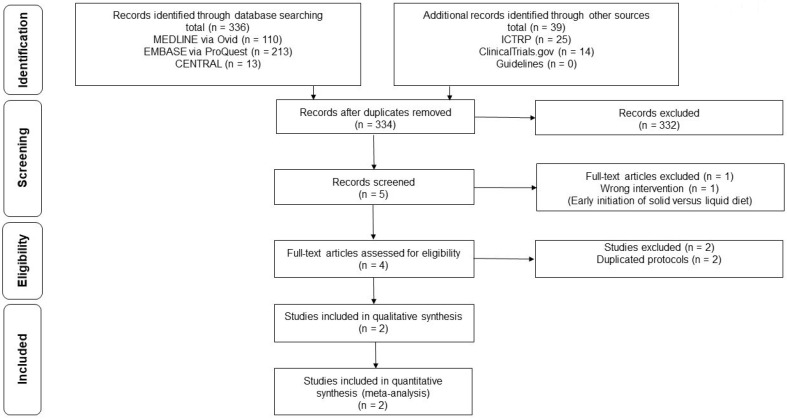
Low diagram of the literature search results.

Figure 1 shows the flow of the study selection of studies comparing early vs. delayed feeding after ESD. The total of 375 records included 5 records after the initial screening. After full-text screening, one article was excluded because this article focused on early initiation of solid and liquid diet [28]. After removing two duplicated protocols, two RCTs (239 patients) were finally included [29,30]. Table 1 summarizes and describes the characteristics of eligible studies. In one study, the early feeding group began feeding on day 0 [29]; in the other study, early feeding started feeding on day 1 after ESD [30]. In two studies, the delayed feeding groups started feeding on day 2 after ESD [29,30]. The RCTs included in this review showed no significant differences in clinicopathological characteristics. Table 2 and Appendix C show the risk of bias in each study. The overall risk of bias for post-ESD bleeding was low in the two studies.

### 3.2. Primary Outcomes

**Figure 2:**
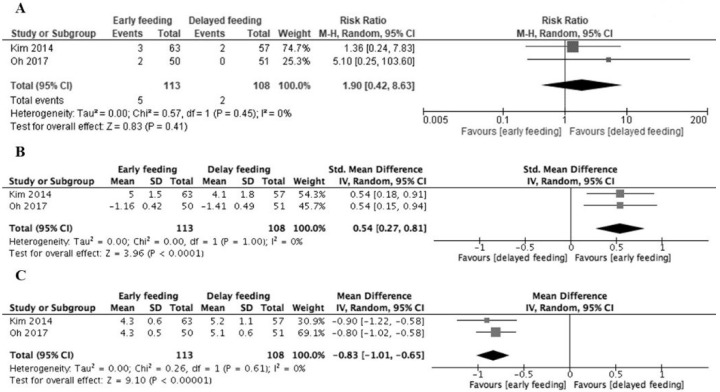
Forest plots. (**A**) Post-endoscopic mucosal resection bleeding. (**B**) Patient satisfaction. (**C**) Length of hospital stay (days).

It is likely that early feeding after ESD was not associated with increased bleeding in comparison to delayed feeding (RR 1.90, 95% CI 0.42 to 8.63; I^2^ = 0%) (Figure 2A). Early feeding after ESD reduced patient dissatisfaction in comparison to delayed feeding (SMD 0.54, 95% CI 0.27 to 0.81; I^2^ = 0%) (Figure 2B). In the early and delayed feeding groups, the mean length of hospital stay was 4.3 and 5.2 days, respectively. Early feeding after ESD resulted in a reduction in the lengths of stay in comparison to delayed feeding (MD −0.83, 95% CI −1.01 to −0.65; I^2^ = 0%) (Figure 2C). The prespecified subgroup analysis and sensitivity analyses on primary outcomes could not be performed when only using data described in the original paper.

### 3.3. Secondary Outcomes

**Figure 3:**
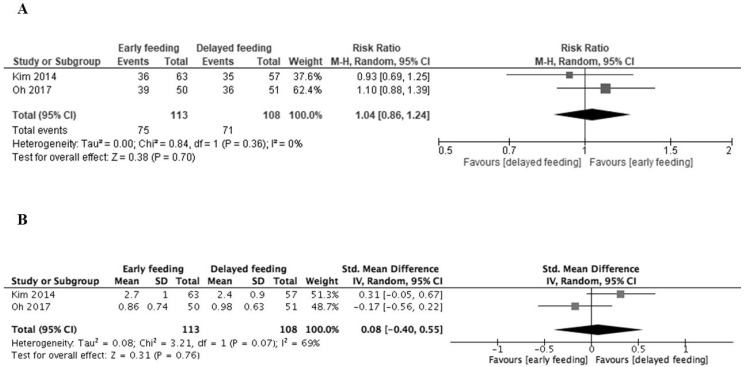
Forest plots. (**A**) Post-ESD ulcer healing status. (**B**) Abdominal pain.

Early feeding after ESD likely made little to no difference on post-ESD ulcer healing in comparison to delayed feeding (RR 1.04, 95% CI 0.86 to 1.24; I^2^ = 0%) (Figure 3A). Early feeding after ESD did not increase abdominal pain in comparison to delayed feeding (SMD 0.08, 95% CI −0.40 to 0.55; I^2^ = 69%) (Figure 3B). One study reported perforation during ESD in one patient (0.7%) before randomization [29], and another reported perforation during ESD in two patients (1.8%) assigned to the early feeding group [30]. In the early feeding group, one patient had dyspnea. In the delayed feeding group, one patient had dementia and one patient had chest pain [30].

### 3.4. Certainty of Evidence

The certainty of evidence was moderate for bleeding and post-ESD ulcer healing, as a result of imprecision due to the small sample size. The certainty of evidence was low as a result of imprecision due to the small sample size and the risk of bias was high because the assessment was likely influenced by knowledge of the intervention (Table 3).

## 4. Discussion

In the present systematic review and meta-analysis, early feeding after ESD was associated with higher patients’ satisfaction and a shorter hospital stay in comparison to delayed feeding after ESD. Furthermore, the early feeding group showed less post-ESD complications. On the other hand, we must acknowledge the limited number of articles included in this review, even though the results from the integrated RCTs on early feeding after ESD may be useful for evidence-based ESD-related practice.

Starvation or hunger will reduce a patient’s satisfaction [31]. Early feeding can also induce early recovery of the intestinal function, which is related to better appetite and bowel peristalsis [28]. These were speculated to be, in part, involved in higher patient satisfaction. Both RCTs in the present review used numeric rating scales to assess patients’ satisfaction [29,30], which included various elements. For instance, the QLQ-C30 and QLQ-STO22 questionnaires on gastric cancer [32,33,34], and the GHAA-9 questionnaire [35] or the State-Trait Anxiety Index [36] have been used to assess the QOL after gastric surgery. Comparable methods may be needed to assess satisfaction in the future.

The short hospital stays observed with early feeding might be explained by the lower rate of post-ESD complications and early oral energy intake that positively contribute to the recovery of physical conditions [28,37]. Shortening the hospital stays with early discharge also seemed to be cost-effective [30]. Previous studies showed that early feeding was less expensive after gastric surgery [38,39]. In a previous RCT, early feeding for 4.3 days of hospitalization was associated with a reduction of the total hospital expense by US$ 385.4 in comparison to delayed feeding for 5.2 days after ESD [30]. This also may lead to an improvement in the patient’s QOL [15,16].

Early feeding did not increase the incidence of post-ESD bleeding. While early feeding appears to induce bleeding, early feeding can neutralize gastric acid secretion, which may suppress bleeding [28]. Furthermore, early feeding did not increase abdominal pain or suppress ulcer healing. This may be partly related to the neutralization of gastric acid secretion with early feeding, as it is known that post-ESD gastric acid secretion induces abdominal pain [40] and ulcers [28]. Additionally, the proton pump inhibitor agents also promote ulcer healing [41,42]. The use of these inhibitors for 8 weeks in the RCTs included in this review was not associated with a difference in abdominal pain or the ulcer status between the early and delayed feeding groups.

The present review was associated with several limitations. First, the number of studies included in the review was relatively small. Second, the risk of bias for outcomes other than post-ESD bleeding and post-ESD ulcer was high. This might be due to the fact that the assessors were not blinded. Third, three patients with perforation during ESD were excluded from the final analysis in the RCTs [29,30]. Such exclusion criteria may produce a registration bias in RCTs unlike real daily practice. More large-scale RCTs with blinded assessors are needed.

In conclusion, the present systematic review and meta-analysis demonstrated that early feeding within 2 days after ESD can have a greater impact on quality of care than delayed feeding. As we must acknowledge the limited number of reviewed studies, various trials regarding the quality of care are further required to establish the benefits of early feeding after ESD.

## Figures and Tables

**Table 1:** Summary of the characteristics of the eligibility studies.

Authors [ref Number]	Year	Subject Number	Age (Years)	Follow-Up (Months)	Initiation of Feeding	Female (%)	Antrum Location of Lesion (%)	Tumor Size (mm)	Total Procedure Time (min)
Kim [29]	2014	120	61.8	2	Early	30.2	68.3	13.1	52.9
					Delayed	26.3	66.7	15.0	61.8
Oh [30]	2017	101	65.9	2	Early	40.0	54.0	15.0	38.1
					Delayed	37.3	66.7	14.8	39.4

**Table 2:** Quality scores for the eligibility studies.

Authors [ref Number]	Risk of Bias 2 Tool Assessment					
	Bias Arising from the Randomization Process	Bias Due to Deviations from Intended Interventions	Bias Due to Missing Outcome Data	Bias in Measurement of the Outcome	Bias in Selection of the Reported Results	Overall Risk of Bias
Kim [29]	Low	Low	Low	Low	Low	Low
Oh [30]	Low	Low	Low	Low	Low	Low

**Table 3:** Summary of findings.

Early vs. Delayed Feeding after Endoscopic Submucosal Dissection						
Patient or Population: Adults Setting: after Endoscopic Submucosal Dissection Intervention: Early Feeding Comparison: Delayed Feeding						
Outcomes	Anticipated Absolute Effects * (95% CI)		Relative Effect (95% CI)	Patient Number (Studies)	Certainty of the Evidence (GRADE)	Comments
	Risk with Delayed Feeding	Risk with Early Feeding				
Bleeding follow-up: within 2 months	20 per 1000	38 per 1000 (8 to 171)	RR 1.90 (0.42 to 8.63)	221 (2 RCTs)	Moderate ^a^	Early feeding likely dose not increase post-ESD bleeding.
Patients’ Satisfaction Assessed with the numeric rating scale	-	MD 0.54 SD higher (0.27 to 0.81)	-	221 (2 RCTs)	Low ^a,b^	The evidence suggests early feeding increases patient satisfaction slightly.
Hospital stay	The mean hospital stay was 5 days	MD 0.83 day lower (−1.01 to −0.65)	-	221 (2 RCTs)	Low ^a,b^	The evidence suggests that early feeding prolong length of stay slightly.
Post-ESD ulcer healing status Follow up: at 2 months	628 per 1000	653 per 1000 (540 to 779)	RR 1.04 (0.86 to 1.24)	221 (2 RCTs)	Moderate ^a^	Early feeding probably results in little to no difference in post-ESD ulcer healing.
Abdominal pain assessed with the numeric rating scale	-	SMD 0.08 SD higher (−0.4 to 0.55)	-	221 (2 RCTs)	Low ^a,b^	The evidence suggests that early feeding does not increase abdominal pain.

CI, confidence interval; MD, mean difference; RR, risk ratio; SMD, standard mean difference. * The risk in the intervention group (and its 95% CI) is based on the assumed risk in the comparison group and the relative effect of the intervention (and its 95% CI). GRADE Working Group grades of evidence; High certainty: We are very confident that the true effect lies close to that of the estimated effect. Moderate certainty: We are moderately confident in the estimated effect. The true effect is likely to be close to the estimated effect, but there is a possibility that it is substantially different. Low certainty: Our confidence in the estimated effect is limited: The true effect may be substantially different from the estimated effect. Very low certainty: We have very little confidence in the estimated effect. The true effect is likely to be substantially different from the estimated effect. ^a^ Downgraded because of imprecision due to the small sample size. ^b^ Downgraded because of high risk of bias because assessment was likely influenced by knowledge of intervention.

## Appendix A. The Electronic Database Search Strategy

**Table A1:** CENTRAL search strategy.

1	MeSH descriptor: [Stomach Neoplasms] explode all trees
2	(gastr$ or stomach): ab,ti,kw
3	(polyp$ or neoplas$ or tumour$ or tumor$ or adenom$ or lesion$ or carcinom$ or adenocarcinom$ or cancer$): ab,ti,kw
4	#2 AND #3
5	#1 OR #4
6	MeSH descriptor: [Endoscopic Mucosal Resection] explode all trees
7	((endoscopic mucosal resection) OR (endoscopic submucosal dissection) OR (ESD) OR (EMR)): ab,ti,kw
8	#6 OR #7
9	#5 AND #8
10	MeSH descriptor: [Diet] explode all trees
11	((feed$) OR (fast$) OR (diet$) OR (intake$)):ab,ti,kw
12	#10 OR #11
13	#9 AND #12

**Table A2:** MEDLINE via Ovid search strategy.

1	exp stomach neoplasms/
2	(gastr$ or stomach).tw.
3	(polyp$ or neoplas$ or tumour$ or tumor$ or adenom$ or lesion$ or carcinom$ or adenocarcinom$ or cancer$).tw.
4	2 and 3
5	1 or 4
6	exp endoscopic mucosal resection/
7	(endoscopic mucosal resection OR endoscopic submucosal dissection OR EMR OR ESD).tw.
8	6 or 7
9	5 and 8
10	exp diet/
11	((feed$) OR (fast$) OR (diet$) OR (intake$)).tw.
12	10 or 11
13	9 and 12

**Table A3:** EMBASE via ProQuest Dialog search strategy.

S1	(EMB.EXACT.EXPLODE (“stomach cancer”))
S2	(ab(gastric) OR ti(gastric) OR ab(stomach) OR ti(stomach))
S3	(ab(polyp) OR ti(polyp) OR ab(polyps) OR ti(polyps) OR ab(neoplasm) OR ti(neioplasm) OR ab(neoplasms) OR ti(neioplasms) OR ab(tumour) OR ti(tumour) OR ab(tumours) OR ti(tumours) OR ab(tumor) OR ti(tumor) OR ab(tumors) OR ti(tumors) OR ab(adenoma) OR ti(adenoma) OR ab(adenomas) OR ti(adenomas) OR ab(lesion) OR ti(lesion) OR ab(lesions) OR ti(lesions) OR ab(carcinoma) OR ti(carcinoma) ab(carcinomas) OR ti(carcinomas) OR ab(adenocarcinoma) OR ti(adenocarcinoma) OR ab(adenocarcinomas) OR ti(adenocarcinomas) OR ab(cancer) OR ti(cancer) OR ab(cancers) OR ti(cancers))
S4	S2 AND S3
S5	S1 OR S4
S6	(EMB.EXACT.EXPLODE (“endoscopic mucosal resection”))
S7	(ab (endoscopic mucosal resection) OR ti(endoscopic mucosal resection) OR ab(endoscopic submucosal dissection) OR ti(endoscopic submucosal dissection) OR ab(ESD) OR ti(ESD) OR ab(EMR) OR ti(EMR))
S8	S6 OR S7
S9	S5 AND S8
S10	(EMB.EXACT.EXPLODE(“dietary intake”))
S11	(ab(feed) OR ti(feed) OR ab(feeding) OR ti(feeding) OR ab(fast) OR ti(fast) OR ab(fasting) OR ti(fasting) OR ab(diet) OR ti(diet) OR ab(intake) OR ti(intake))
S12	S10 OR S11
S13	S9 AND S12

## Appendix B. The Trial Registry Search Strategy

ICTRP search strategy

(“endoscopic mucosal resection” OR “endoscopic submucosal dissection” OR “EMR” OR “ESD”) AND (“feed” OR “fast” OR “diet” OR “intake”)

ClinicalTrials.gov search strategy

Other terms: (“endoscopic mucosal resection” OR “endoscopic submucosal dissection” OR “EMR” OR “ESD”) AND (“feed” OR “fast” OR “diet” OR “intake”)

## Appendix C. Quality Scores for the Eligibility Studies

**Table A4:** Patients’ satisfaction.

**Authors** **[ref no.]**	**Risk of Bias 2 Tool Assessment**					
	Bias Arising from the Randomization Process	Bias Due to Deviations from Intended Interventions	Bias Due to Missing Outcome Data	Bias in Measurement of the Outcome	Bias in Selection of the Reported Results	Overall Risk of Bias
Kim [29]	Low	Low	Low	High	Low	High
Oh [30]	Low	Low	Low	High	High	High
Length of stay						
Kim [29]	Low	Low	Low	High	Low	High
Oh [30]	Low	Low	Low	High	Low	High
Post-ESD ulcer healing status						
Kim [29]	Low	Low	Low	Low	Low	Low
Oh [30]	Low	Low	Low	Low	Low	Low
Abdominal pain						
Kim [29]	Low	Low	Low	High	High	High
Oh [30]	Low	Low	Low	High	Low	High
